# Anterior Tooth Inclination Between Skeletal Class II and III Malocclusions After Surgical Orthodontic Treatment

**DOI:** 10.3390/diagnostics15121553

**Published:** 2025-06-18

**Authors:** Hiromi Tomaru, Chie Tachiki, Yu Nakamura, Dai Ariizumi, Satoru Matsunaga, Keisuke Sugahara, Akira Watanabe, Akira Katakura, Yasushi Nishii

**Affiliations:** 1 Department of Orthodontics, Tokyo Dental College, Chiyoda, Tokyo 101-0061, Japan; wakamatsuhiromi@tdc.ac.jp (H.T.); ariizumidai@tdc.ac.jp (D.A.); nishii@tdc.ac.jp (Y.N.); 2 Kawasemi Dental Clinic, Funabashi 273-0005, Chiba, Japan; nakamurayuu@icloud.com; 3 Department of Anatomy, Tokyo Dental College, Chiyoda, Tokyo 101-0061, Japan; matsuna@tdc.ac.jp; 4 Department of Oral Pathobiological Science and Surgery, Tokyo Dental College, Chiyoda, Tokyo 101-0061, Japan; ksugahara@tdc.ac.jp (K.S.); katakura@tdc.ac.jp (A.K.); 5 Department of Oral & Maxillofacial Surgery, Tokyo Dental College, Chiyoda, Tokyo 101-0061, Japan; akirawat@tdc.ac.jp

**Keywords:** surgical orthodontics, cephalometric analysis, anterior tooth inclination, skeletal malocclusion

## Abstract

**Background/Objectives:** Anterior tooth inclination plays a critical role in treatment planning for surgical orthodontic cases. However, post-treatment outcomes in patients with jaw deformities often deviate from cephalometric values. This study aimed to compare anterior tooth inclination and skeletal morphology among patients with Class II and Class III malocclusions and to establish reference values for individualized treatment plans. **Methods**: A total of 122 patients (Class II: *n* = 40; Class III: *n* = 41; Class I: *n* = 41 as a control) were retrospectively analyzed. Cephalometric parameters, including U1 to FH and L1 to MP, were measured pre- and post-treatment. Group differences were analyzed using one-way ANOVA and Tukey’s multiple comparison test. Multiple regression analysis was used to establish prediction models for anterior tooth inclination. The threshold for statistical significance was set at *p* < 0.05. **Results**: Post-treatment, upper anterior teeth were more lingually inclined in Class II patients and more labially inclined in Class III patients compared to Class I controls (U1 to FH: Class II, 106.8°; Class III, 120.4°; Class I, 111.1°; *p* < 0.01). Lower anterior teeth were more lingually inclined in Class III patients compared to Class I patients (L1 to MP: 84.9°; *p* < 0.01). Regression models demonstrated good predictive value (R^2^ > 0.5) in non-extraction cases. **Conclusions**: Regression equations developed in this study, alongside the cephalometric averages of Class I individuals, may offer reliable reference values tailored to individual craniofacial morphology, contributing to optimized treatment planning in surgical orthodontic cases.

## 1. Introduction

The objective of surgical orthodontic treatment is to achieve optimal skeletal and dental relationships based on the cephalometric norms derived from individuals with normal occlusion. However, in clinical practice, the outcomes of patients with jaw deformities do not always conform to these standard ranges. Among the various treatment considerations, the appropriate axial inclination of the upper and lower anterior teeth is particularly important for orthodontists when planning preoperative orthodontic interventions [1]. Anterior tooth inclination has been shown to influence not only esthetics and occlusion but also long-term periodontal health [2,3].

Skeletal Class II and Class III malocclusions are characterized not only by differences in horizontal jaw relationships but also by variations in vertical skeletal morphology. Skeletal Class II cases are frequently associated with high-angle patterns, whereas Class III cases often exhibit low-angle configurations [4,5]. These morphological differences may influence the extent of vertical surgical movement required, as well as the long-term stability of treatment outcomes [6,7,8,9,10,11,12,13,14,15]. Although the ideal treatment goal is theoretically to achieve skeletal Class I relationships, differences in muscular function and skeletal structure between Class II and III may render this approach suboptimal in certain cases [16,17,18].

While previous studies have reported cephalometric changes before and after surgical orthodontic treatment, few have directly compared skeletal morphology and anterior tooth inclination post-treatment among Class II and Class III patients [19,20,21]. Moreover, there is a lack of objective, quantitative indices that aid in establishing individualized treatment targets, especially concerning anterior tooth inclination after orthognathic surgery. This is a critical gap, given the potential risks associated with improper decompensation, including alveolar bone loss and gingival recession [22,23,24].

We hypothesized that the skeletal morphology following surgical orthodontic treatment significantly differs between skeletal Class II and Class III patients and that these morphological differences result in significantly different axial inclinations of the anterior teeth.

Therefore, the present study aimed to compare skeletal discrepancies and anterior tooth inclination between patients with skeletal Class II and Class III morphologies, focusing on cases that demonstrated stable occlusion after surgical orthodontic treatment. Furthermore, this study sought to propose target values for skeletal positioning and anterior tooth inclination based on regression analyses that account for individual craniofacial morphology.

## 2. Materials and Methods

### 2.1. Subjects and Materials

This was a retrospective, single-center cohort study conducted at Suidobashi Hospital, Tokyo Dental College. A total of 40 patients with skeletal Class II malocclusion (Class II group) and 41 patients with skeletal Class III malocclusion (Class III group) who received treatment at the Department of Orthodontics were included. The control group (Class I group) consisted of 41 randomly selected patients with skeletal Class I malocclusion who had undergone orthodontic treatment. All patients were diagnosed with jaw deformities and underwent surgical orthodontic treatment. Only those who exhibited stable occlusion at two years post-retention and had complete permanent dentition, excluding third molars, were included. Furthermore, the cephalograms used for analysis were taken immediately after orthodontic treatment to evaluate the final treatment outcome. Patients with a history of prior orthodontic treatment were excluded. Lateral cephalometric radiographs obtained before (pre-treatment) and after (post-treatment) surgical orthodontic treatment were used for analysis.

The inclusion criteria were an ANB angle of 5° or greater and a Wits appraisal of +2 mm or greater for the Class II group and an ANB angle of 0° or less and a Wits appraisal of −6 mm or less for the Class III group. Additional skeletal criteria included an SNA angle of 80° or greater for Class II and 84° or less for Class III and an SNB angle of 76° or less for Class II and 86° or greater for Class III. For the control group, patients with an ANB angle between 2° and 4°, a Wits appraisal between −5 mm and +1 mm, an SNA angle between 80° and 84°, and an SNB angle between 76° and 86° for Class I were included. Molar relationships corresponded to the skeletal classifications Class I, II, and III. These parameters were selected for their diagnostic reliability in detecting skeletal Class II and skeletal Class III, respectively [25,26,27] (Table 1).

Patients in each group (Class I, Class II, and Class III) were further categorized into extraction and non-extraction subgroups. In the extraction group, four first premolars (maxillary and mandibular bilaterally) were extracted in both Class I and Class II cases. In Class III cases, two maxillary first premolars were bilaterally extracted. Extraction protocols were determined at the beginning of presurgical orthodontic treatment, based on factors such as dental crowding, incisor inclination, and the requirements for surgical decompensation.

Exclusion criteria included patients with congenital anomalies such as cleft lip and palate, severe anterior open bite less than −2 mm, facial asymmetry with Menton deviation exceeding 3 mm, missing or prosthetic upper and lower anterior teeth, multiple missing teeth, a history of genioplasty, or patients treated using a surgery-first protocol (Table 2). For the Class I group, patients exhibiting prominent skeletal discrepancies indicative of Class II or Class III malocclusions were excluded. Specifically, cases with ANB ≥ 5°, ANB ≤ 0°, Wits appraisal ≥ +2 mm, or Wits appraisal ≤ −6 mm were excluded to ensure the exclusion of borderline or misclassified skeletal patterns.

Surgical orthodontic treatment was performed by nine orthodontists, all certified specialists of the Japanese Orthodontic Association with more than 10 years of clinical experience. The treatment followed the standard protocol covered by Japanese health insurance, and surgery-first approaches were excluded. Orthognathic surgery was performed by oral surgeons. To minimize variation in treatment outcomes, all patients were treated according to standardized surgical and orthodontic protocols, including consistent use of model surgery, identical bracket systems, and controlled archwire sequences. Only cases undergoing SSRO alone or Le Fort I + SSRO were included to further reduce procedural variability. Preoperative orthodontic treatment was carried out using a multi-bracket system (0.022-inch slot, pre-adjusted edgewise brackets, OPA-K®, Tomy International Inc., Shinagawa, Tokyo, Japan) with 0.019″ × 0.025″ stainless steel archwires as working wires. After the completion of preoperative orthodontic treatment, orthognathic surgery was performed according to the surgical treatment plan, which was developed based on cephalometric prediction and model surgery. The surgical procedures included either sagittal split ramus osteotomy (SSRO) alone or bimaxillary surgery combining Le Fort I osteotomy and SSRO. Bone fixation was achieved by semi-rigid fixation using either titanium or absorbable mini-plates. Postoperative orthodontic treatment commenced once patients achieved adequate mouth opening, and retention was maintained using circumferential and fixed-type retainers in the upper and lower jaws after the completion of active treatment. Patient characteristics and treatment breakdowns are presented in Table 3.

### 2.2. Cephalometric Landmarks and Measured Values

**Figure 1:**
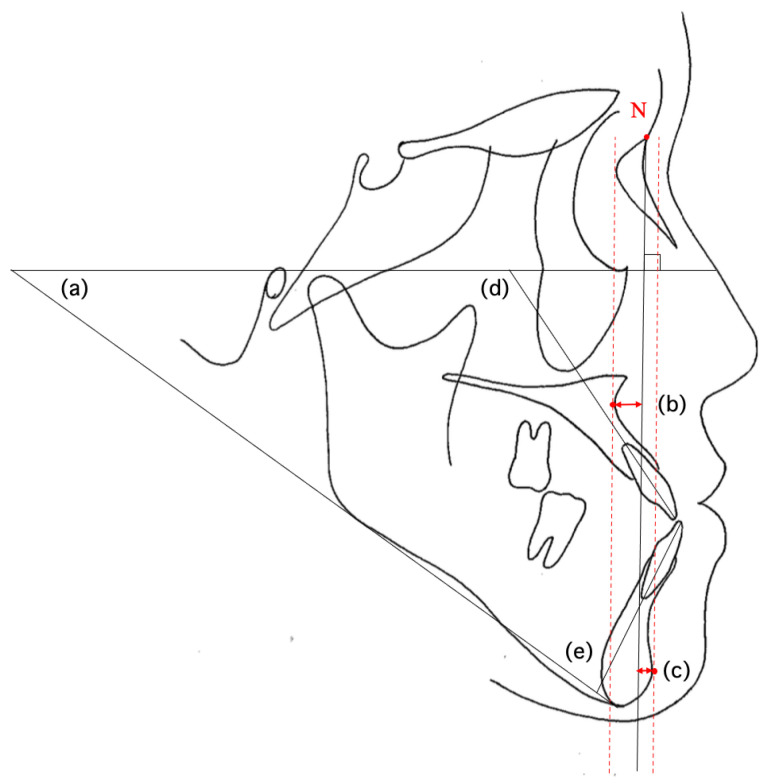
Measurement items in multiple regression analysis: (**a**) FMA; (**b**) Mc PtA; (**c**) Mc Pog; (**d**) U1 to FH; (**e**) L1 to MP. The black line extending from “N” represents the McNamara line. The red dotted lines indicate perpendiculars from Point A and Pogonion to the McNamara line. “N” indicates Nasion.

Cephalometric tracings were prepared from the lateral cephalometric radiographs of each subject. Measurements were performed based on predefined skeletal landmarks, tooth axes, and soft tissue reference points (Table 4, Figure 1). The inclination of anterior teeth was measured using U1 to the Frankfort horizontal plane (U1-FH) and L1 to the mandibular plane (L1-MP), which are standard cephalometric indicators of axial inclination. Tracing and measurements were performed by a single orthodontist with five years of clinical experience and were independently verified by a second orthodontist, who served as an advisor with 16 years of experience. One of the supervisors was a clinical instructor and board-certified specialist of the Japanese Orthodontic Society.

To assess intra-examiner reliability, three repeated measurements were conducted by the same orthodontist, and the intraclass correlation coefficient (ICC) was calculated. The ICC value for intra-examiner reliability was 0.90, indicating minimal measurement error. Inter-examiner reliability was assessed by calculating the ICC between the two examiners, resulting in a value of 0.92, demonstrating a high level of agreement. Dahlberg’s error between the first and second measurements was 0.3° for Class I, 0.3° for Class II, and 0.3° for Class III. Cronbach’s alpha values were 0.993 (Class I), 0.985 (Class II), and 0.995 (Class III), confirming excellent internal consistency. These results confirm the high reliability and reproducibility of the measurements. All measurements were performed using Quick Ceph Studio (version 5.0.2, Quick Ceph Systems, San Diego, CA, USA), which was also utilized in planning the surgical procedure in collaboration with the oral surgeons.

### 2.3. Statistical Processing

Sample size was calculated using G*Power (version 3.1.9.6 for Windows) based on our preliminary investigation. Effect size (f^2^ = 0.20) was assumed, with a significance level (α) of 0.05 and statistical power (1 − β) of 0.80. With three predictors in the multiple linear regression model, the minimum required sample size was calculated to be 59.

Therefore, a total of 61 participants (20 or 21 per group) was considered sufficient. This estimate was based on previous studies analyzing cephalometric changes in surgical orthodontic patients [28,29]. Furthermore, random errors were estimated using the Dahlberg formula.

Normality of distribution was assessed using the Shapiro–Wilk test for each cephalometric parameter. While most variables met the assumption of normality (*p* > 0.05), some did not. Homogeneity of variance across the Class I, II, and Class III groups was examined using Levene’s test. The test results indicated that certain variables did not satisfy the assumption of equal variances (*p* < 0.05). Despite these deviations, one-way ANOVA followed by Tukey’s multiple comparison test was employed for intergroup comparisons. This decision was based on the robustness of ANOVA in the presence of moderate violations and the balanced sample sizes across groups. The threshold for statistical significance was set at *p* < 0.05 for all tests.

Additionally, pre-treatment factors influencing the post-treatment inclination of the upper and lower anterior teeth were analyzed for both the Class II and Class III groups. Multiple regression analysis with a stepwise selection method was used to predict the post-treatment anterior tooth inclination for the Class II and Class III groups. To evaluate the impact of premolar extraction, multiple regression analyses were separately performed for the extraction and non-extraction groups. Statistical analyses were performed using SPSS software (version 27.0; IBM Corp., Armonk, NY, USA).

### 2.4. Clinical Application Procedure

To evaluate the clinical applicability of the multiple regression equations developed in this study, four post-surgical orthodontic treatment cases that were not involved in the creation of the regression models but met the inclusion criteria were randomly selected. The multiple regression equations were then applied to these cases, and the predicted postoperative axial inclinations of the upper and lower anterior teeth were compared with the actual measured values. This approach was used to assess the clinical applicability of the equations.

## 3. Results

### 3.1. Comparison of Cephalometric Values Between the Three Groups

Table 5 presents the comparison of mean cephalometric values among the Class I, Class II, and Class III groups at pre-treatment. In comparisons between the Class I and Class II groups, the Class II group exhibited significantly higher values for ANB, Wits appraisal, FMA, overjet, and upper and lower lip positions relative to the E-line. Conversely, the Class II group demonstrated significantly lower values for Mc Pog and U1 to FH (*p* < 0.05).

In the comparison between the Class I and Class III groups, the Class III group exhibited significantly higher values for Mc Pog while showing significantly lower values for ANB, Wits appraisal, L1 to MP, overjet, and upper and lower lip positions relative to the E-line (*p* < 0.05).

Comparison between the Class II and Class III groups revealed that the Class II group exhibited significantly higher values for Mc Pog and U1 to FH. On the other hand, the Class II group showed significantly lower values for ANB, Wits appraisal, FMA, L1 to MP, overjet, and upper and lower lip positions relative to the E-line (*p* < 0.05).

Table 6 summarizes the cephalometric values of each group at post-treatment. The results demonstrated that even after treatment, significant differences in skeletal and dental measurements persisted between the groups, with skeletal Class II and Class III patients exhibiting distinctive dental compensation patterns when compared with skeletal Class I patients.

### 3.2. Examination of Pre-Treatment Factors Affecting the Upper and Lower Anterior Tooth Inclination Post-Treatment in Class II and Class III Groups

As the stepwise regression method demonstrated low predictive accuracy (R^2^ < 0.5) for the extraction group, the analysis focused on the non-extraction group, where higher predictive validity was observed.

#### 3.2.1. Class II Group

For the Class II non-extraction group, the dependent variable was U1 to FH (post-treatment), with U1 to FH (pre-treatment), Mc Pog (pre-treatment), and FMA (pre-treatment) as independent variables. Multiple regression analysis demonstrated that Mc Pog (pre-treatment) was a significant predictor, with a standardized partial regression coefficient of 0.726 (Table 7). U1 to FH (pre-treatment) and FMA (pre-treatment) were excluded by the stepwise selection method. The R² value of 0.527 indicated that the model was valid.

Similarly, for L1 to MP (post-treatment), the dependent variable was L1 to MP (pre-treatment), with Mc Pog (pre-treatment) and FMA (pre-treatment) as independent variables. The standardized partial regression coefficient of L1 to MP (pre-treatment) was 0.708, indicating a strong influence (Table 8). Mc Pog (pre-treatment) and FMA (pre-treatment) were excluded by the stepwise method. The R² value of 0.501 indicated that the model was valid.

R^2^ = 0.527

Regression equation:

U1 to FH (post) = 117.143 + 0.453 × Mc Pog (pre)

R^2^ = 0.501

Regression equation:

L1 to MP (post) = 19.413 + 0.804 × L1 to MP (pre)

#### 3.2.2. Class III Group

In the Class III non-extraction group, for U1 to FH (post-treatment), the standardized partial regression coefficients for U1 to FH (pre-treatment) and Mc Pog (pre-treatment) were 0.445 and 0.404, respectively, indicating moderate influence (Table 9). FMA (pre-treatment) was excluded by the stepwise selection method. The R² value of 0.531 indicated that the model was valid.

For L1 to MP (post-treatment), the standardized partial regression coefficients for L1 to MP (pre-treatment) and FMA (pre-treatment) were 0.679 and 0.420, respectively, suggesting strong and moderate influence (Table 10). Mc Pog (pre-treatment) was excluded by the stepwise selection method. The R² value of 0.508 indicated that the model was valid.

R^2^ = 0.531

Regression equation:

U1 to FH (post) = 60.030 + 0.472 × U1 to FH (pre) + 0.658× Mc Pog (pre)

R^2^ = 0.508

Regression equation:

L1 to MP (post) = 8.467 + 0.787 × L1 to MP (pre) + 0.405 × FMA (pre)

### 3.3. Results of Clinical Application

**Figure 2:**
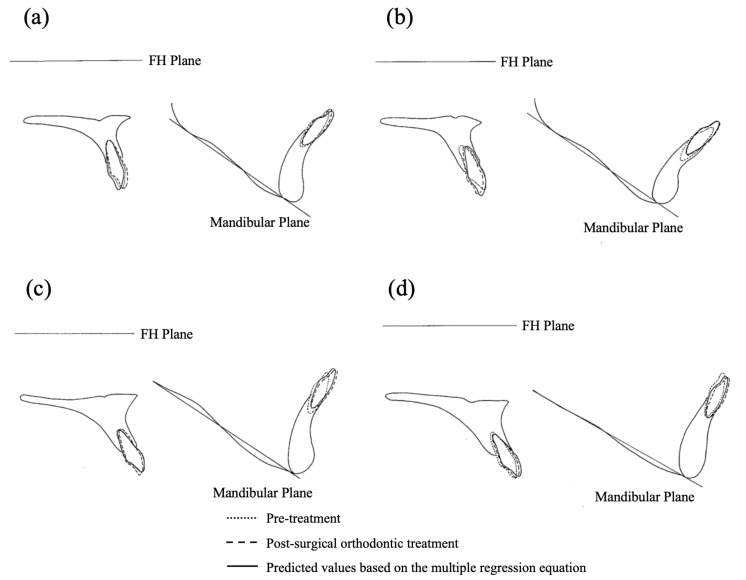
Four cases used for the external check of the clinical application of the multiple regression equation of this study. Overlaid cephalometric tracings of the upper and lower jaw in pre-treatment, post-treatment, and prediction by multiple regression equations: (**a**) Case 1 (skeletal Class II); (**b**) Case 2 (skeletal Class II); (**c**) Case 3 (skeletal Class III); (**d**) Case 4 (skeletal Class III).

As an external validation, the multiple regression equations developed in this study were applied to four additional cases (Case 1, Case 2, Case 3, and Case 4) that were not included in the generation of the regression models (Figure 2). This approach was used to assess the clinical applicability of the equations in predicting the postoperative axial inclinations of the upper and lower anterior teeth.

Table 11 and Table 12 present the predicted and actual measured postoperative values for these four cases. The residuals between the predicted and measured values for U1 to FH were slightly larger in Case 1 and Case 2, ranging from ±1.8° to 3.2°. However, for all other measurements, the residuals were within ±0.3° to 1.3°, indicating good predictive accuracy of the regression equations.

These findings suggest that the regression equations may provide reliable estimates for the postoperative axial inclinations of the anterior teeth, supporting their potential utility in clinical orthodontic treatment planning.

## 4. Discussion

### 4.1. Comparison of Cephalometric Analysis Items Between the Three Groups Before and After Treatment

Potts et al. reported that, in skeletal Class II patients undergoing surgical orthodontic treatment, the upper anterior teeth exhibited lingual inclination preoperatively and tended to return toward a normal position postoperatively, whereas the lower anterior teeth were labially inclined preoperatively, with dental compensation persisting after treatment [30]. Consistent with these findings, the present study demonstrated that the cephalometric measurements of skeletal Class II patients after surgical orthodontic treatment remained different from those of skeletal Class I patients. These results suggest that, in skeletal Class II patients, postoperative skeletal positioning often retains Class II characteristics, accompanied by residual dental compensation of the upper and lower anterior teeth.

In skeletal Class III patients, previous studies reported that the postoperative upper anterior teeth exhibited labial inclination, while the lower anterior teeth remained lingually inclined, indicating that dental compensation was not fully eliminated [31]. In the present study, cephalometric analysis of skeletal Class III patients after treatment similarly demonstrated persistence of anterior chin positioning, labial inclination of the upper anterior teeth, and lingual inclination of the lower anterior teeth, underscoring the importance of evaluating residual dental compensation.

These findings provide important reference data for establishing target values of anterior tooth inclination in surgical orthodontic treatment. Achieving appropriate anterior tooth inclination before surgery significantly contributes to accurate skeletal repositioning, minimizes residual dental compensation, and enhances long-term functional stability and esthetic harmony after orthognathic treatment [20]. The persistence of dental compensation after treatment highlights the necessity of detailed treatment planning that considers individual skeletal morphology rather than solely relying on standard skeletal Class I values.

### 4.2. Examination of Pre-Treatment Factors Affecting the Upper and Lower Tooth Inclination

The findings of this study revealed that dental compensation persisted in the upper and lower anterior teeth after treatment in both skeletal Class II and Class III patients. These results suggest that the axial inclinations of anterior teeth following surgical orthodontic treatment in patients with jaw deformities differ from the normative values observed in skeletal Class I malocclusions.

In skeletal Class II patients treated with orthodontic camouflage alone, excessive labial inclination of the lower anterior teeth may increase the risk of long-term periodontal complications, including bone loss and gingival recession [32]. In skeletal Class III patients, the alveolar bone surrounding the lower anterior teeth is also lingually inclined and tends to be thinner compared to that in skeletal Class I patients [33,34,35,36,37]. Therefore, in patients with mild skeletal discrepancies, orthodontic treatment alone may be sufficient to achieve improved alignment by adjusting the axial inclinations of the anterior teeth. However, in patients with moderate to severe skeletal discrepancies, correction of skeletal deformity through surgical orthodontic intervention is recommended to achieve stable occlusion and satisfactory facial aesthetics.

Treatment planning for surgical orthodontics often uses the cephalometric mean values of individuals with normal occlusion as reference targets. However, morphological and functional differences in the cranio-maxillofacial hard and soft tissues are frequently observed in patients with jaw deformities compared to those with skeletal Class I. Moreover, the skeletal outcomes of Class II and Class III patients after treatment do not consistently fall within the normative range.

Particular attention should be paid to the inclination of the anterior teeth during preoperative orthodontic treatment [20]. Ma et al. emphasized that teeth should be maintained within the alveolar bone envelope during preoperative orthodontic treatment to avoid risks such as alveolar fenestration and dehiscence [21]. In severe skeletal Class III patients, complete dental decompensation based on conventional incisor angulation criteria may result in the anterior teeth exceeding the limits of the alveolar bone, leading to unfavorable outcomes such as gingival recession or bone loss [23,24,31,34,38,39,40]. Therefore, the use of more appropriate indices for predicting and evaluating anterior tooth inclination, such as the regression equations developed in this study, may help achieve safer and more effective treatment planning.

While several previous studies have reported on soft tissue profile prediction and skeletal changes after surgical orthodontic treatment [41,42,43,44,45], few have focused on establishing specific target values for anterior tooth inclination [46]. The reliable results of the multiple regression analyses conducted in this study, particularly for non-extraction cases, suggest that these equations may serve as useful tools for setting treatment targets in surgical orthodontics.

### 4.3. Evaluation of the Research Hypothesis

The hypothesis proposed in this study was that the axial inclinations of the anterior teeth following surgical orthodontic treatment significantly differ between skeletal Class II and Class III patients and are influenced by specific pre-treatment skeletal parameters.

Our results support this hypothesis. The post-treatment anterior tooth inclinations systematically varied between the skeletal Class II and Class III groups, with distinctive patterns of dental compensation observed in each group. Furthermore, multiple regression analyses identified key pre-treatment skeletal parameters—particularly Mc Pog and FMA—that significantly contributed to predicting the final inclination of both upper and lower anterior teeth. The predictive equations developed in this study exhibited high explanatory power (R^2^ > 0.5) and were successfully validated using external clinical cases, further confirming the hypothesis.

These findings underscore the importance of individualized treatment planning based on skeletal morphology rather than uniform reliance on skeletal Class I norms.

### 4.4. Clinical Application

The multiple regression equations derived from this study demonstrated good predictive accuracy and clinical applicability. These findings suggest that the anterior–posterior positioning of both the upper and lower anterior teeth significantly influences their postoperative axial inclination. By integrating these regression equations into treatment planning, orthodontists may be able to achieve more accurate and stable tooth positioning in surgical orthodontic cases.

External validation using four additional cases confirmed the practical utility of the regression models in predicting postoperative anterior tooth inclination. These results support the potential role of these equations in improving treatment outcomes through individualized planning based on each patient’s skeletal morphology.

### 4.5. Study Limitations

A primary limitation of this study is that the sample population exclusively consisted of Japanese patients. Therefore, further studies including participants from diverse ethnic backgrounds are necessary, as skeletal and dental morphology may vary across different populations.

Additionally, cephalometric analyses were conducted only at the pre-treatment and post-treatment stages. Performing evaluations at multiple time points—including pre-treatment, post-preoperative orthodontic treatment, immediately post-surgery, and final post-treatment—would provide a more detailed understanding of tooth movement and skeletal changes throughout the course of treatment. Future research incorporating these additional time points would more comprehensively help clarify the dynamics of skeletal and dental changes.

Another limitation of this study is that the assumptions of normality and homogeneity of variances were not fully satisfied for all variables, as confirmed by the Shapiro–Wilk and Levene’s tests. While one-way ANOVA was used as the primary method of analysis, future research should consider using Welch’s ANOVA or non-parametric alternatives, such as the Kruskal–Wallis test, particularly for variables showing significant distributional irregularities.

## 5. Conclusions

This study revealed that skeletal Class II malocclusions exhibit greater lingual inclination of the upper anterior teeth, while skeletal Class III malocclusions show labial inclination of the upper and lingual inclination of the lower anterior teeth. These findings reflect characteristic dental compensation patterns that remain even after surgery. Therefore, in treatment planning for Class II and III malocclusions, it is advisable to consider not only the cephalometric norms of skeletal Class I malocclusions but also the regression equations derived in this study, which offer individualized normative values. Applying these models may enhance the accuracy of treatment planning and contribute to more stable and esthetically favorable outcomes.

## Figures and Tables

**Table 1:** Inclusion criteria.

	Inclusion Criteria		
Parameter	Class II	Class III	Class I
ANB angle	ANB ≥ 5°	ANB ≤ −1°	0° < ANB < 5°
Wits appraisal	Wits ≥ +2 mm	Wits ≤ −6 mm	−6 < Wits < 2 mm
SNA angle	SNA ≥ 80°	SNA ≤ 84°	80° < SNA < 84°
SNB angle	SNB ≤ 76°	SNB ≥ 86°	76° < SNB < 86°
Molar relationship	Angle Class II	Angle Class III	Angle Class I molar relationship or mild deviations

**Table 2:** Exclusion criteria.

Parameter	Exclusion Criteria
Congenital anomalies	Cleft lip and palate
Anterior open bite	Overbite ≤ −2 mm
Facial asymmetry	Menton deviation > 3 mm
Missing or prosthetic anterior teeth	Patients with one or more missing or prosthetic anterior teeth
Multiple missing teeth	Patients with three or more missing permanent teeth (excluding third molars)
History of genioplasty	History of genioplasty
Surgery-first protocol	Cases treated with surgery-first approach

**Table 3:** Patient characteristics of each group at pre-treatment.

	Class II Group (ANB ≥ 5°) *n* = 40	Class III Group (Wits ≤ −6 mm) *n* = 41	Class I Group (0° < ANB < 5°) *n* = 41
Sex			
Male	10	17	12
Female	30	24	29
Age (y)			
Mean	29.7 ± 8.4	29.6 ± 7.5	20.5 ± 8.0
Range	15.0–49.0	18.6–51.8	11.0–58.5
Ext or Non Ext			
Ext	20	21	28
Non Ext	20	20	13
Orthognathic surgery			
SSRO	20	21	
Le Fort I + SSRO	20	20	

**Table 4:** Measurement items in multiple regression analysis.

Parameter	Definition
FMA	The angle formed between the Frankfort horizontal plane and the mandibular plane
Mc PtA	The distance between the nasion perpendicular line and point A, measured perpendicular to the nasion perpendicular line
Mc Pog	The distance between the pogonion and the nasion perpendicular line measured perpendicular to the nasion perpendicular line
U1 to FH	The angle between the long axis of the upper central incisor (U1) and the Frankfort horizontal plane
L1 to MP	The angle between the long axis of the lower central incisor (L1) and the mandibular plane

**Table 5:** Comparison of cephalometric values between each group at pre-treatment.

	Class I		Class II		Class III		Cl.I–Cl.II	Cl.I–Cl.III	Cl.II–Cl.III
	Mean	S.D.	Mean	S.D.	Mean	S.D.			
ANB (°)	2.5	1.5	8.6	2.5	−3.0	2.9	**	**	**
Mc PtA (mm)	−1.1	2.7	−0.9	5.3	−0.9	3.5	N.S.	N.S.	N.S.
Mc Pog (mm)	−5.8	5.4	−20.2	12.2	8.1	7.4	**	**	**
Wits (mm)	−1.6	3.4	5.7	6.7	−14.1	5.7	**	**	**
FMA (°)	30.8	5.8	37.9	8.5	28.3	7.2	**	N.S.	**
U1 to FH (°)	116.9	8.4	112.1	9.4	119.5	6.4	*	N.S.	**
L1 to MP (°)	95.5	7.5	98.1	8.6	82.9	8.8	N.S.	**	**
Overjet (mm)	3.2	2.7	7.4	2.7	−2.1	2.7	**	**	**
Overbite (mm)	1.1	1.9	0.1	4.0	0.3	2.0	N.S.	N.S.	N.S.
E-line upper (mm)	−0.2	2.6	3.7	2.8	−3.7	2.6	**	**	**
E-line lower (mm)	1.5	3.1	4.7	3.3	−0.2	3.4	**	*	**

One-way ANOVA and Tukey’s multiple comparison test; * *p* < 0.05; ** *p* < 0.01; N.S. not significant.

**Table 6:** Comparison of cephalometric values between each group at post-treatment.

	Class I		Class II		Class III		Cl.I–Cl.II	Cl.I–Cl.III	Cl.II–Cl.III
	Mean	S.D.	Mean	S.D.	Mean	S.D.			
ANB (°)	2.4	1.5	6.3	2.5	0	2.2	**	**	**
Mc PtA (mm)	−1.4	2.9	−1.3	5.8	−0.3	2.8	N.S.	N.S.	N.S.
Mc Pog (mm)	−6.1	5.4	−14.8	10.9	0.6	7.4	**	**	**
Wits (mm)	−1.5	3.2	2.0	4.2	−6.2	3.5	**	**	**
FMA (°)	30.7	5.6	36.5	8.7	29.3	6.6	**	N.S.	**
U1 to FH (°)	111.1	7.9	106.8	6.9	120.4	7.2	*	**	**
L1 to MP (°)	92.4	7.8	95.8	6.8	84.9	7.3	N.S.	**	**
Overjet (mm)	2.7	0.9	2.8	0.9	3.1	0.8	N.S.	N.S.	N.S.
Overbite (mm)	1.9	0.8	2.1	1.7	1.3	0.8	N.S.	N.S.	*
E-line upper (mm)	−1.0	2.4	1.0	2.8	−2.4	2.1	**	*	**
E-line lower (mm)	0.2	2.1	1.7	2.9	−1.0	2.9	*	N.S.	**

One-way ANOVA and Tukey’s multiple comparison test; * *p* < 0.05; ** *p* < 0.01; N.S. not significant.

**Table 7:** The effect of pre-treatment cephalometric value on post-treatment U1-FH in the Class II/non-extraction group.

	Regression Coefficient	Standardized Regression Coefficient	Significance (*p*)	95% Confidence Interval	
				Lower	Upper
Constant	117.143		0.000 **	112.623	121.663
Mc Pog (pre)	0.453	0.726	0.000 **	0.240	0.665

Multiple regression analysis using the stepwise method. ANOVA *p* < 0.001; ** *p* < 0.01.

**Table 8:** The effect of pre-treatment cephalometric value on post-treatment L1-MP in the Class II/non-extraction group.

	Regression Coefficient	Standardized Regression Coefficient	Significance (*p*)	95% Confidence Interval	
				Lower	Upper
Constant	19.413		0.305	−19.219	58.046
L1-MP (pre)	0.804	0.708	0.000 **	0.406	1.201

Multiple regression analysis using the stepwise method. ANOVA *p* < 0.001; ** *p* < 0.01.

**Table 9:** The effect of pre-treatment cephalometric value on post-treatment U1-FH in the Class III/non-extraction group.

	Regression Coefficient	Standardized Regression Coefficient	Significance (*p*)	95% Confidence Interval	
				Lower	Upper
Constant	60.030		0.017 *	12.385	107.675
U1-FH (pre)	0.472	0.445	0.030 *	0.050	0.895
Mc Pog (pre)	0.658	0.404	0.047 *	0.010	1.306

Multiple regression analysis using the stepwise method. ANOVA *p* < 0.01; * *p <* 0.05.

**Table 10:** The effect of pre-treatment cephalometric value on post-treatment L1-MP in the Class III/non-extraction group.

	Regression Coefficient	Standardized Regression Coefficient	Significance (*p*)	95%Confidence Interval	
				Lower	Upper
Constant	8.467		0.656	−30.888	47.821
L1-MP (pre)	0.787	0.679	0.001 **	0.360	1.214
FMA (pre)	0.405	0.420	0.028 *	0.050	0.761

Multiple regression analysis using the stepwise method. ANOVA *p* < 0.01; ** *p* < 0.01; * *p* < 0.05.

**Table 11:** The predicted and measured values of the anterior tooth inclination at post-treatment obtained from multiple regression equations (Class II).

	Case 1			Case 2		
	Measured Value	Predicted Value	Residual	Measured Value	Predicted Value	Residual
U1 to FH	104.0	107.2	−3.2	108.0	106.7	1.3
L1 to MP	95.0	94.2	0.8	100.2	100.5	−0.3

**Table 12:** The predicted and measured values of the anterior tooth inclination at post-treatment obtained from multiple regression equations (Class III).

	Case 3			Case 4		
	Measured Value	Predicted Value	Residual	Measured Value	Predicted Value	Residual
U1 to FH	121.0	120.0	1.0	121.6	119.8	1.8
L1 to MP	84.6	85.9	−1.3	85.1	84.6	0.5

## Data Availability

The data presented in this study are available upon request from the corresponding author. The data are not publicly available due to ethical restrictions and privacy concerns related to patient information.
